# The Bacterial Wilt Reservoir Host *Solanum dulcamara* Shows Resistance to *Ralstonia solanacearum* Infection

**DOI:** 10.3389/fpls.2021.755708

**Published:** 2021-11-10

**Authors:** Pau Sebastià, Roger de Pedro-Jové, Benoit Daubech, Anurag Kashyap, Núria S. Coll, Marc Valls

**Affiliations:** ^1^Centre for Research in Agricultural Genomics (CSIC-IRTA-UAB-UB), Bellaterra, Spain; ^2^Department of Genetics, University of Barcelona, Barcelona, Spain

**Keywords:** bacterial wilt, *Ralstonia solanacearum*, disease resistance, reservoir host plants, vascular reinforcements, overwintering

## Abstract

*Ralstonia solanacearum* causes bacterial wilt, a devastating plant disease, responsible for serious losses on many crop plants. *R. solanacearum* phylotype II-B1 strains have caused important outbreaks in temperate regions, where the pathogen has been identified inside asymptomatic bittersweet (*Solanum dulcamara*) plants near rivers and in potato fields. *S. dulcamara* is a perennial species described as a reservoir host where *R. solanacearum* can overwinter, but their interaction remains uncharacterised. In this study, we have systematically analysed *R. solanacearum* infection in *S. dulcamara*, dissecting the behaviour of this plant compared with susceptible hosts such as tomato cv. Marmande, for which the interaction is well described. Compared with susceptible tomatoes, *S. dulcamara* plants (i) show delayed symptomatology and bacterial progression, (ii) restrict bacterial movement inside and between xylem vessels, (iii) limit bacterial root colonisation, and (iv) show constitutively higher lignification in the stem. Taken together, these results demonstrate that *S. dulcamara* behaves as partially resistant to bacterial wilt, a property that is enhanced at lower temperatures. This study proves that tolerance (i.e., the capacity to reduce the negative effects of infection) is not required for a wild plant to act as a reservoir host. We propose that inherent resistance (impediment to colonisation) and a perennial habit enable bittersweet plants to behave as reservoirs for *R. solanacearum*.

## Introduction

Alternate or reservoir hosts are non-target organisms that can harbour high amounts of pathogens for long periods of time and serve as an inoculum source for further infections on the primary host (Haydon et al., 2002; Morris et al., 2009). However, the term “reservoir host” has been also applied to natural or economically unimportant hosts or to hosts where infections are always non-pathogenic (Haydon et al., 2002). In many important crop diseases, non-agricultural reservoirs have also been proposed to enhance the adaptive potential of pathogens and influence disease epidemiology (Mueller et al., 2012; Monteil et al., 2013; Thinakaran et al., 2015; McCann, 2020). For instance, *Pseudomonas syringae* isolated from wild species was shown to potentially develop into novel crop pathovars in a few evolutionary steps (Monteil et al., 2013; Bartoli et al., 2015), and experimental evolution experiments with *Ralstonia solanacearum* demonstrated high fitness gains when this pathogen was inoculated on distant hosts (Guidot et al., 2014).

*Ralstonia solanacearum* is the agent causing the devastating bacterial wilt disease in over 200 plant species, including economically important crops such as potato, tomato, peanut, eggplant, and banana (Hayward, 1994; Mansfield et al., 2012; Coll and Valls, 2013). *R. solanacearum* can survive in the soil and waterways (Van Elsas et al., 2000; Álvarez et al., 2008a), from where it infects plants through the roots and colonises the xylem tissue, blocking water flow and causing plant wilting (Hayward, 1991; Schell, 2000). The disease is endemic in tropical and subtropical areas, but *R. solanacearum* phylotype II-B1 (formerly race 3 biovar 2) strains are adapted to cooler temperatures and have caused important outbreaks in temperate regions (Elphinstone, 1996; Janse et al., 2004; Champoiseau et al., 2009).

Survival and overwintering of *R. solanacearum* in temperate regions appears to rely on infection of perennial reservoir host plants because its persistence in the soil is limited (Olsson, 1976; Shamsuddin et al., 1978; Elphinstone, 1996). Bittersweet (*Solanum dulcamara*) is a common holarctic perennial weed that has been proposed to play a reservoir role in the persistence and spread of *R. solanacearum* based on several observations. Firstly, common incidences of *R. solanacearum* in *S. dulcamara* have been described along waterways (Kempenaar et al., 1998), and most disease outbreaks were related to watercourses in which infected *S. dulcamara* plants were present (Olsson, 1976; Elphinstone, 1996; Janse et al., 1998). Secondly, winter persistence of *R. solanacearum* in waterways correlated with the presence of the pathogen in *S. dulcamara* plants growing near them (Olsson, 1976; Elphinstone et al., 1998; Caruso et al., 2005). Thirdly, *R. solanacearum* was shown to colonise asymptomatically the roots and vascular tissue of *S. dulcamara* plants in the wild, and infected plants were shown to release the bacterium onto surface water *via* aquatic roots (Olsson, 1976; Elphinstone, 1996; Janse, 1996). Besides *S. dulcamara, R. solanacearum* phylotype IIB sequevar 1 strain have been found growing asymptomatically in the wild with other weeds that act as sources of inoculum to infect potato fields. These include *Solanum nigrum* (Olsson, 1976), *Solanum cinereum* (Graham and Lloyd, 1978), *Urtica dioica* in Europe (Wenneker et al., 1999), and a number of wild species from the Ugandan highlands (Tusiime et al., 1997). In China, tropical *R. solanacearum* strains were also identified in the weed *Ageratum conyzoides* L., often showing wilting symptoms (She et al., 2013).

The interactions between *R. solanacearum* and its cultivated hosts have been well-characterised, but little is known about the behaviour of this pathogen inside wild hosts. *R. solanacearum* inoculation on *S. dulcamara* in laboratory conditions has been previously reported (Wenneker et al., 1999; Álvarez et al., 2008b; Jacobs et al., 2013). A first assay screening a large number of plant species found that 66% of *S. dulcamara* plants inoculated through soil drenching became infected (Álvarez et al., 2008b). However, the authors classified this plant as tolerant to bacterial wilt because colonisation was only apparent in 25% of the plants, in which the bacterium occupied a few xylem vessels or occasionally all xylem bundles (Álvarez et al., 2008b). In another report, wilting was more apparent after soil drench inoculations, and *S. dulcamara* plants showed intermediate symptomatology compared with susceptible (Bonny Best) and resistant (Hawaii 7996) tomato plants (Jacobs et al., 2013). In a third study, all plants became infected and 97% showed symptoms when the bacterium was directly inoculated in the stem. However, symptomatology and pathogen presence was restricted to inoculated shoots, indicating slow or no spreading of the bacterium throughout the plant (Wenneker et al., 1999). In this same study, only 13–19% of the plants were infected and 9% showed symptoms when plants were soil-drench inoculated (Wenneker et al., 1999). In summary, *S. dulcamara* presents highly variable symptomatology in response to *R. solanacearum* depending on the inoculation method, although it usually shows an intermediate behaviour between a susceptible and a resistant host. The mechanisms responsible for this partial restriction of colonisation by *R. solanacearum* have not yet been described.

In this study, we have undertaken a thorough characterisation of the interaction between *R. solanacearum* and its wild host *S. dulcamara*. We describe the localisation of the pathogen during the infection process and the symptomatology on the plant at different temperatures and compare this interaction with that established on susceptible tomato (cv. Marmande) and potato (cv. Desirée) plants.

## Materials and Methods

### Plant and Bacterial Materials and Growth Conditions

Bittersweet (*S. dulcamara*) plants were grown from seeds harvested from wild specimens in Vidrà (NE Catalonia, Spain). The susceptible tomato (*Solanum lycopersicum* cv. Marmande) and susceptible potato (*Solanum tuberosum* cv. Desirée) plants used in this study are commercially available.

For pot experiments, *S. dulcamara* and *S. lycopersicum* cv. Marmande seeds were surface-sterilised in 35% bleach and 0.02% Triton-X 100 for 10 min and then rinsed with sterile distilled water five times before sowing them in soil (Substrate 2, Klasmann-Deilmann GmbH) mixed with perlite and vermiculite (30:1:1) and grown under controlled conditions for 3 weeks under a long-day photoperiod (16 h light/8 h dark) and under a light intensity of 120–150 μmol⋅m^–2^⋅ s^–1^ at 22°C and 60% humidity. For optimal germination, *S. dulcamara* seeds were stratified at 4°C for 2 weeks before transferring them to 22°C. *S. tuberosum* cv. Desirée potato plants were propagated *in vitro* (Puigvert et al., 2017) and 2-week old apex was sown in the same soil mixture described above and grown in the same conditions.

All infection assays were performed using the *R. solanacearum* strain UY031 (phylotype IIB, sequevar 1) isolated from potato tubers in Uruguay (Siri et al., 2011), carrying either the synthetic *luxCDABE* operon or the GFPuv gene, both under the control of the constitutive *psbA* promoter (Monteiro et al., 2012). Bacteria were routinely grown at 28–30°C in rich B medium in liquid cultures supplemented with gentamicin (10 μg/ml) and the same medium, supplemented with 0.5% glucose and 50 mg/l of triphenyl tetrazolium chloride for growth on semi-solid agar plates (Monteiro et al., 2012).

### Plant Inoculation and Pathogenicity Assays

For soil-soaking and stem inoculation assays, plants were grown for 3–4 weeks. Soil-soaking root inoculations were performed by pouring 40 ml of 10^8^ colony-forming units (CFU)⋅ml^–1^ (*OD*_600_ = 0.1) of bacterial suspension on every plant pot without disturbing the roots. Infected plants were kept in a growth chamber set at 27°C (exceptionally 20°C when indicated) and scored for wilting symptoms using a scale from 0 to 4, 0 = healthy plant with no wilt, 1 = 25%, 2 = 50%, 3 = 75% of the leaves wilted, and 4 = total wilting. Disease indexes were calculated by averaging the disease score of each plant of the experiment (*n* > 15) as indicated in previous publications with *S. dulcamara*, tomato, and potato (Álvarez et al., 2008b; Siri et al., 2011; Planas-Marquès et al., 2020). Stem-inoculation assays were performed by applying a 5 μl droplet of a 10^6^ CFU⋅ml^–1^ (*OD*_600_ = 0.001) bacterial solution twice with a sterile 0.3 × 13 mm needle (30GX 1/2″, BD Microlance, Becton Dickinson) to the wounds caused at the base of the petiole after removal of the first true leaf. After inoculation, plants were kept in a growth chamber set at 27°C unless otherwise specified and scored for wilting symptoms as described before (Monteiro et al., 2012).

To quantify the bacterial content inside the shoots, 2 cm sections were excised from above the taproot (soil-soaked plants) or above the inoculation point (stem-inoculated plants), weighed, and incubated for at least 30 min in a sterile 2-ml Eppendorf tube with 300 μl of sterile distilled water to let the bacterium ooze from the tissue. Luminescence was measured from the tubes containing excised tissue with a luminometer (FB 12, Berthold Detection Systems) to determine the bacterial concentrations since luminescence was proven to strongly correlate with bacterial density (Planas-Marquès et al., 2020). To measure bacterial counts in the root, plants inoculated as described were uprooted from day 1 to day 4 post-inoculation, and the roots were rinsed with distilled water. Approximately 1–2 cm of root below the tap root were cut and ground. Tissue was weighed and CFUs were counted as described above. Dilution plating of samples on rich B medium and CFU counting 24 h later was performed in some cases to verify luminescence results.

### Assessment of Bacterial Colonisation

Plant colonisation by *R. solanacearum* was assessed using the luminescent and fluorescent strains described above. Plant stems inoculated with the luminescent strain were sliced using a sterile razor blade obtaining internode sections just below and above the petiole where inoculation had been carried out. One millimetre thick transversal cuts and 1 cm long longitudinal cuts were placed flat on a square plate and visualised using a live imaging system (ChemiDoc Touch Imaging System, Bio-Rad) using a 5-min exposure time with 3 × 3 sensitivity. Images were processed using Image Lab software (Bio-Rad). Soil-soak inoculated plants with the luminescent strain were photographed by placing the whole plant in a Fuji Film LAS4000 light imager system with a 15-min exposure time.

Stem-inoculated plants with the fluorescent strain were dissected as described before and photographed using binocular microscopy equipped with a UV fluorescent lamp (BP330-385 BA420 filter) and an SZX16 stereomicroscope equipped with a DP71 camera system (Olympus) using the following settings: GFP filter, 10 s exposure time, ISO 1/800. Soil-soaked plants with the fluorescent strain were photographed with a Leica DM6 microscope. Bright field or fluorescence images merging the UV channel for plant structures (blue) and the GFP channel for bacteria (green) were automatically assembled by the microscope software to obtain single images including the whole root section.

Quantification of the black signal (luminescence) or the green channel (fluorescence) in the pictures was quantified using the Fiji software (United States National Institutes of Health).

### Tissue Stainings

After producing root wounds with a 1 ml pipette tip, the plants were soil-soaked with a bacterial solution of 10^7^ CFU⋅g^–1^ (*OD*_600_ = 0.01). The day the plants showed an adequate disease index, the taproots were transversally sliced. Then four to five slices per plant were placed in a 1.5-ml tube with 70% ethanol for at least 7 days to remove the chlorophyll. For lignin staining, individual taproot slices were placed on a microscope slide and incubated with two drops of phloroglucinol HCl for about 1 min, then rinsed with 70% ethanol, and a cover slide was placed on top for visualisation in the upright microscope (Leica DM6) bright-field (Pomar et al., 2004). Mock-infected plants were inoculated with water. Lignin quantification was performed by selecting the vascular area in tomato and potato plants and comparing it with the same area in *S. dulcamara* plants using ImageJ software. Images were converted to a greyscale (eight-bit image) and the mean grey value was calculated.

For suberin staining, individual *S. dulcamara*, tomato cv. Marmande and potato cv. Desirée taproot slices were placed in a well containing a Sudan IV solution for 5 min and then rinsed in another well with 70% ethanol as described (Kashyap et al., 2021). Clean slices were placed on a slide and visualised with the UV filter on a Leica DM6 microscope.

### Statistical Analyses

Statistical analyses were performed using Statgraphics software. All statistical tests are indicated in the respective figure legends.

## Results

### *Solanum dulcamara* Shows an Enhanced Capacity to Withstand *R. solanacearum* Infection in Comparison With Tomato cv. Marmande

**FIGURE 1:**
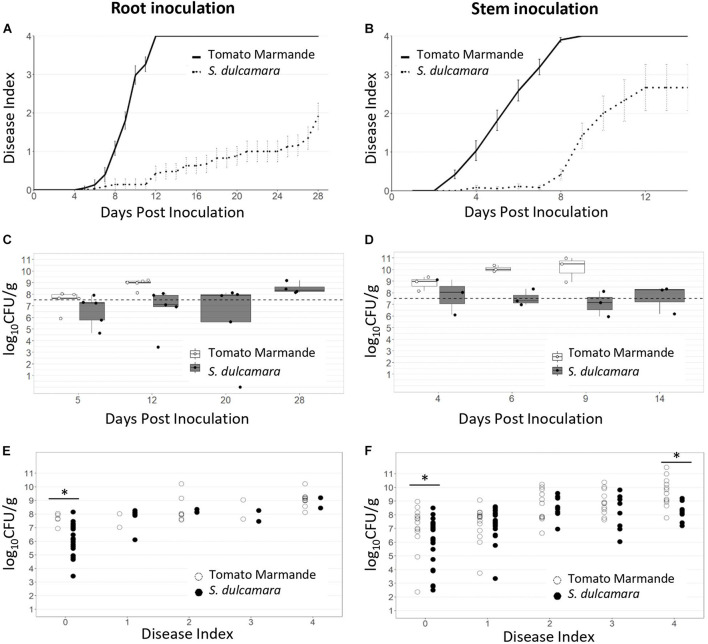
Bacterial wilt evaluation in *S. dulcamara* and tomato cv. Marmande plants. Plants of the wild reservoir host *S. dulcamara* and tomato susceptible to bacterial wilt were root inoculated by soil soaking **(A,C,E)** or stem inoculated **(B,D,F)** with *R. solanacearum* UY031 carrying a luminescent reporter. **(A,B)** Wilting symptoms were recorded over time using a scale from 0 (no wilting) to 4 (completely wilted). *n* = 30–35 plants per plant species. **(C,D)** Bacterial concentrations in the stem at different time points from plants in panels **(A,B)**, respectively. *n* = 4–8 plants per sampling day. **(E,F)** Bacterial content in relation to wilting symptoms for each plant individual analysed of the two species. *n* = 30 plant samples analysed for each species. Bacterial counts are expressed as log CFUs⋅g^–1^ tissue. ^∗^indicates statistical differences (*p value* < 0.05, *T*-student significant test). The experiments in panels **(A,C)** were repeated three times with similar results. The experiment in panels **(B,D)** was repeated twice with similar results.

To analyse the symptomatology caused by *R. solanacearum* in *S. dulcamara* and compare its behaviour with that of tomato, we inoculated plants in controlled conditions using two different methods. First, we used a more naturalistic root inoculation method by soaking the soil with a bacterial solution without causing any wounding to plants, after which the plants were kept at 27°C, and then the wilting symptoms were recorded over time. All susceptible tomato Marmande plants were completely wilted 14 days post-inoculation (dpi), while the symptoms just started to appear in *S. dulcamara* (Figure 1A). By 28 dpi, less than half of the *S. dulcamara* plants had completely wilted, showing a clear delay in the development of the disease with respect to tomato plants (*p-value* < 0.0001; Figure 1A).

The second method used was direct bacterial inoculation inside the plant stem vasculature, a more aggressive procedure that skips the first infection steps (root entry and vascular colonisation). As expected, disease progression was faster after stem inoculation and all the tomato plants were completely wilted at 8 dpi. *S. dulcamara* plants still showed a clear delay in disease progression after stem inoculation (*p-value* < 0.0001): first symptoms were apparent only by day 8, although most plants were completely wilted by 14 dpi (Figure 1B). Interestingly, an important proportion of *S. dulcamara* plants remained asymptomatic at the end of our experiments, especially when soil inoculation was performed. Quantification of bacterial loads in the stem over time showed an overall correlation with disease symptoms (Figures 1C,D). In soil-drench inoculations, when tomato plants were almost completely wilted (12 dpi), the bacterial concentrations in their stems were ∼10^9^ CFU⋅g^–1^, significantly higher than the ∼10^7^ CFU⋅g^–1^ found in *S. dulcamara*, which only showed minor symptoms at this time point (Figure 1C). Similar results with bacterial concentrations were obtained in stem inoculation experiments although these plants displayed higher bacterial contents, especially at late disease stages, since structural barriers present in roots are circumvented in this inoculation method (Figure 1D).

To precisely determine the bacterial concentrations that the two plant species could withstand inside their tissues, we took the data from all biological replicas and plotted bacterial content in relation to wilting symptoms for each plant analysed. This representation clearly showed that, irrespective of the inoculation method, *S. dulcamara* and susceptible tomato bore similar bacterial concentrations at intermediate wilting stages (Figures 1E,F). However, two clear differences were observed: (i) as hinted before, at early disease stages (disease index = 1) *S. dulcamara* plants showed lower bacterial colonisation, and (ii) bacterial loads rarely exceeded 10^9^ CFU⋅g^–1^ in *S. dulcamara*, whereas they often overcame these levels in tomato plants, leading to statistical differences when plants were completely wilted. The differences in the late disease stages were more apparent in stem inoculation experiments because this more aggressive inoculation method resulted in a higher proportion of plants showing symptoms and becoming totally wilted (disease index 4) compared with natural root inoculations by soil drenching (Figures 1E,F). In summary, *S. dulcamara* plants displayed delayed bacterial wilt symptom development compared with susceptible tomato plants, with most of the individuals surviving infection in the timeframe of our experiments. In addition, lower *R. solanacearum* concentrations were observed in the stems of *S. dulcamara* at early and late disease stages, suggesting delayed colonisation and restriction to bacterial growth.

### A High Proportion of *S. dulcamara* Plants Show Long-Lasting Latent Infections

**FIGURE 2:**
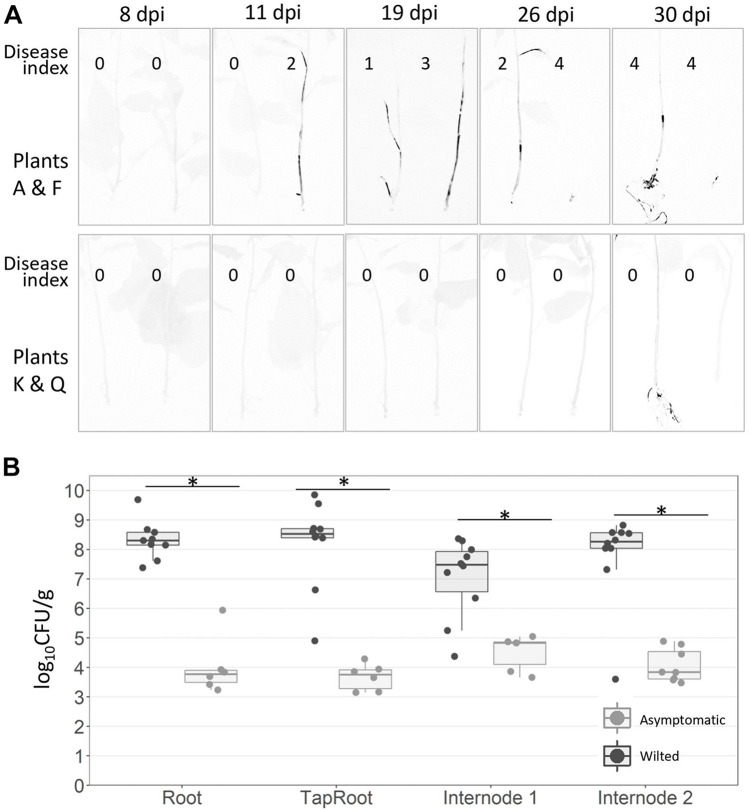
Bacterial colonisation and multiplication in stem-inoculated *S. dulcamara* and tomato cv. Marmande plants. **(A)** Non-destructive live luminescence imaging of four representatives *S. dulcamara* plants throughout a 30-day period after root inoculation with a luminescent *R. solanacearum* strain. Two symptomatic (A & F) and two asymptomatic (K & Q) plants are shown. Luminescent bacteria are detected in darker areas. Wilting symptoms (Disease index = 0–4) are indicated next to each plant inside the images. **(B)** *R. solanacearum* concentrations measured at the root, taproot, internode 1 (2–3 cm above the inoculation point) and 2 (6–9 cm above the inoculation point) in *S. dulcamara* plants uprooted 30 days post-inoculation (dpi) with the luminescent reporter strain. The results from asymptomatic (disease index 0) and symptomatic (disease index 1–4) plants are shown separately. Bacterial counts were calculated from tissue luminescence and are expressed as log CFUs⋅g^–1^ tissue. ^∗^indicates statistical differences (*p*-value < 0.05, *T*-student significant test).

*Ralstonia solanacearum* infection heterogeneity amongst different plant individuals is common. To analyse the progression of bacterial colonisation and disease symptoms in single plants over time, we took advantage of the luminescent *R. solanacearum* strain used in this work, which could be visualised non-destructively inside plant tissues (Cruz et al., 2014). Stem inoculations were used in these experiments to reduce the high stochasticity of root inoculations and to facilitate infection so that a significant proportion of plants become completely wilted. Live imaging and symptom recording of whole plants were carried out along a 30-day period, after which the plants were uprooted to visualise bacterial content in the roots. As observed before, in this experiment, half of the *S. dulcamara* plants showed symptoms, and half of them remained asymptomatic at the end of the assay (Figure 2A and Supplementary Figure 1). Bacterial colonisation paralleled the onset of disease symptoms in wilting plants (Figure 2A top panel) and was always undetectable in the aerial tissues of asymptomatic plants (Figure 2A bottom panel). Interestingly, *R. solanacearum* latent infections were detected in the most asymptomatic plants (four out of six plants Supplementary Figure 1), which displayed detectable luminescence in the root at 30 dpi (Figure 2A bottom panel and Supplementary Figure 1). Quantification of the black signal from the pictures in S1 showed that the area colonised by bacteria positively correlates with the disease symptoms, except in totally wilted plants where tissue collapse and drying causes bacterial death (Supplementary Figure 2). For a more sensitive and quantitative analysis, the bacterial contents of root and stem sections of plants uprooted at 30 dpi were calculated. The results proved that *R. solanacearum* was present in all tissues analysed from asymptomatic *S. dulcamara* plants, although bacterial concentrations were in almost all cases four orders of magnitude lower than that in symptomatic plants (Figure 2B).

In summary, long-term challenging of *S. dulcamara* with *R. solanacearum* always resulted in two distinct behaviours: plants with apparent bacterial colonisation and disease symptoms and plants that remained symptomless even after direct stem inoculation, but which always carried latent bacterial infections.

### *Ralstonia solanacearum* Movement Is Restricted in *S. dulcamara* Tissues Compared With Susceptible Tomato cv. Marmande

**FIGURE 3:**
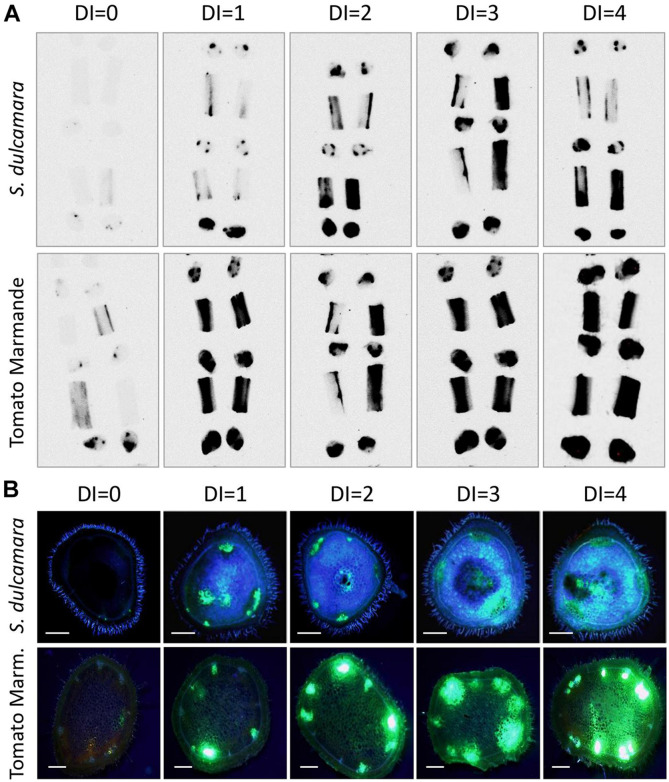
*R. solanacearum* distribution in stems of inoculated *S. dulcamara* and tomato cv. Marmande plants. **(A)** Representative luminescence imaging photographs at different wilting stages (Disease index 0–4) of stem sections from *S. dulcamara* (top panel) and tomato (bottom panel) plants stem-inoculated with luminescent *R. solanacearum*. Luminescent bacteria are detected as dark areas in transversal and longitudinal sections of plant internodes 1–4 organised bottom to top. Inoculation points are indicated by an arrow. **(B)** Representative fluorescence microscopy images of stem sections from *S. dulcamara* (top panel) and tomato (bottom panel) plants stem-inoculated with an *R. solanacearum* strain constitutively expressing GFP. Inoculations were performed at the base of the first true leaf and transversal sections obtained in the first internode, 2 cm above the inoculation point. Scale bars indicate 0.5 mm.

We have previously demonstrated that resistant tomato varieties can restrict *R. solanacearum* root colonisation and hamper bacterial vertical and horizontal movements in the stem (Planas-Marquès et al., 2020). Thus, we hypothesised that this mechanism could be also active in *S. dulcamara* and cause the observed delay in symptom appearance and infection latency. Next, we evaluated if *S. dulcamara* plants restricted bacterial movement in the stems compared with susceptible tomatoes. To better compare bacterial behaviour in the two hosts, we stem-inoculated a large number of plants with the luminescent reporter strain and observed bacterial distribution in their stems by grouping the plants according to disease stage. The whole 4-to-5-week-old plants could not be imaged because of size limitations and reduced sensitivity due to stem thickness. Thus, we obtained stem sections of internodes one to four from plants and imaged the top and bottom slices of each section and the remaining stem longitudinally divided in two. Representative pictures presented in Figure 3A show that luminescence matched the location of xylem bundles and was less intense in *S. dulcamara* plants compared with tomatoes at early disease stages. Quantification of the luminescence signal (Supplementary Figure 3A) corroborated this result, supporting the lower bacterial loads previously observed in asymptomatic *S. dulcamara* (Figures 1E,F). In addition, the luminescence of xylem bundles tended to decrease with height in *S. dulcamara*, while it remained constant in the susceptible tomato plants (Figure 3A), suggesting stronger restriction to vertical bacterial movement along the vessels in *S. dulcamara*.

To further analyse if *S. dulcamara* restricts the horizontal spread of *R. solanacearum* to neighbouring xylem and parenchyma tissues, we observed shoot sections of plants stem-inoculated with a GFP-tagged strain (Cruz et al., 2014) using fluorescence microscopy. Representative images of internode cuts above the inoculation point showed that the bacterium was slightly more confined to the vasculature in *S. dulcamara*, and that a lower number of xylem vessels appeared fluorescent in this species with respect to tomato plants at comparable disease stages (Figure 3B). Despite the differences observed in asymptomatic plants, quantification of the fluorescence intensity in diseased plants (Supplementary Figure 3B) showed that colonisation was comparable in tomato and *S. dulacamara* plants displaying similar symptomatology.

Since stem inoculation skips the initial stages of infection, and to determine whether *R. solanacearum* root entry and colonisation were also restricted in *S. dulcamara* plants, we carried out a root inoculation experiment. Briefly, plants were inoculated with the luminescent reporter strain by soil drenching and the bacterial counts were measured at short times after inoculation (1–4 dpi). As can be observed in Supplementary Figure 4, bacterial concentrations were comparable at early time points, demonstrating no difference in root entry. However, statistically lower bacterial loads were observed in *S. dulcamara* roots at 4 dpi, proving that the root tissues of *S. dulcamara* also limit *R. solanacearum* colonisation.

Taken together, the assessment of bacterial colonisation in shoots and roots of both hosts suggests that *S. dulcamara* plants cope better with bacterial wilt because they have the ability to effectively restrict pathogen movement and colonisation inside their tissues.

### *Solanum dulcamara* Displays Dramatically Reduced Bacterial Wilt Symptoms and Bacterial Colonisation at 20°C

**FIGURE 4:**
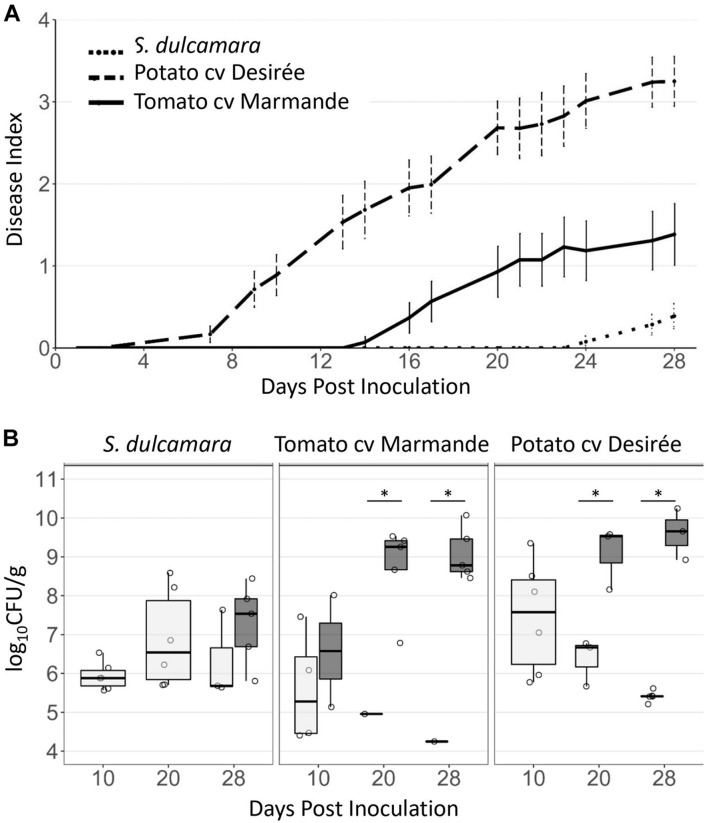
Influence of temperature on bacterial wilt symptomatology in *S. dulcamara* and susceptible tomato and potato varieties. **(A)** Bacterial wilt symptom development (0 = asymptomatic to 4 = completely wilted) after soil-drench inoculation in *S. dulcamara*, tomato cv. Marmande and potato cv. Desirée plants inoculated with the *R. solanacearum* luminescent reporter and kept at 20°C. *n* = 30–35 plants per species. **(B)** Bacterial concentrations at the taproot level quantified by measuring the luminescence at 10, 20 and 28 dpi from *S. dulcamara*, susceptible tomato cv. Marmande, and susceptible potato cv. Desirée inoculated as in panel **(A)**. Results are grouped according to disease symptoms: asymptomatic (grey, disease index 0) and symptomatic (white, disease indices 1–4). *n* = 4–8 plants per sampling day and condition. ^∗^indicates statistical differences (*p* value < 0.05, *T*-student significant test).

*Solanum dulcamara* has been demonstrated to be a reservoir plant host in which *R. solanacearum* can overwinter (Olsson, 1976; Elphinstone et al., 1998; Janse et al., 1998; Wenneker et al., 1999; Caruso et al., 2005). To test the plant behaviour at lower temperatures that mimic those encountered in temperate environments, *S. dulcamara* and tomato plants kept at 20°C were soil-soak inoculated with luminescent *R. solanacearum*, and the symptoms and bacterial loads in the stems were evaluated over time. A temperature of 20°C was chosen as the lower temperature, compared with 27°C to avoid strong effects on plant or pathogen growth. To rule out specific effects of lower temperatures on the tomato-control plants, susceptible potato plants (cv. Desirée), which are adapted to cooler conditions than tomatoes (Ingram and McCloud, 1984), were also included in this experiment. Few days after inoculation, the first tomato plants started to wilt, followed by the first potato plants 2 weeks after inoculation. By 30 dpi, around 50% of the potatoes and over 25% of the tomatoes were completely wilted (Figure 4A), in accordance with previous results in tomatoes inoculated at these temperatures with a closely related II-B1 strain (Milling et al., 2009). On the contrary, all *S. dulcamara* plants survived the infection at 30 dpi with only a few of them (six out of 25) showing mild wilting symptoms in individual leaves (disease index <0.5, Figure 4A). Quantification of bacterial levels in the stem over time correlated with wilting, showing overall lower bacterial titres in *S. dulcamara* than in susceptible tomato or potato plants (Figure 4B). Since most plants remained asymptomatic throughout the experimental period, bacterial concentrations were calculated separately for asymptomatic and symptomatic plants. Symptomatic plants carried bacterial counts above 10^7^ CFUs⋅g^–1^ in all species, concentrations being the lowest in *S. dulcamara* because disease symptoms were less developed in this species compared with the two susceptible crops. For instance, 30 days after inoculation *R. solanacearum* counts reached a maximum of 5 × 10^8^ CFUs⋅g^–1^ in *S. dulcamara*, whereas wilted potato and tomato plants harboured up to 10^10^ CFUs⋅g^–1^ (Figure 4B).

In conclusion, cooler temperatures slowed down disease development in all species, but this effect was more pronounced in *S. dulcamara*, which always survived the disease in the tested period while holding mostly asymptomatic (latent) bacterial infections.

### *Solanum dulcamara* Contains a Constitutively and Highly Lignified Xylem

**FIGURE 5:**
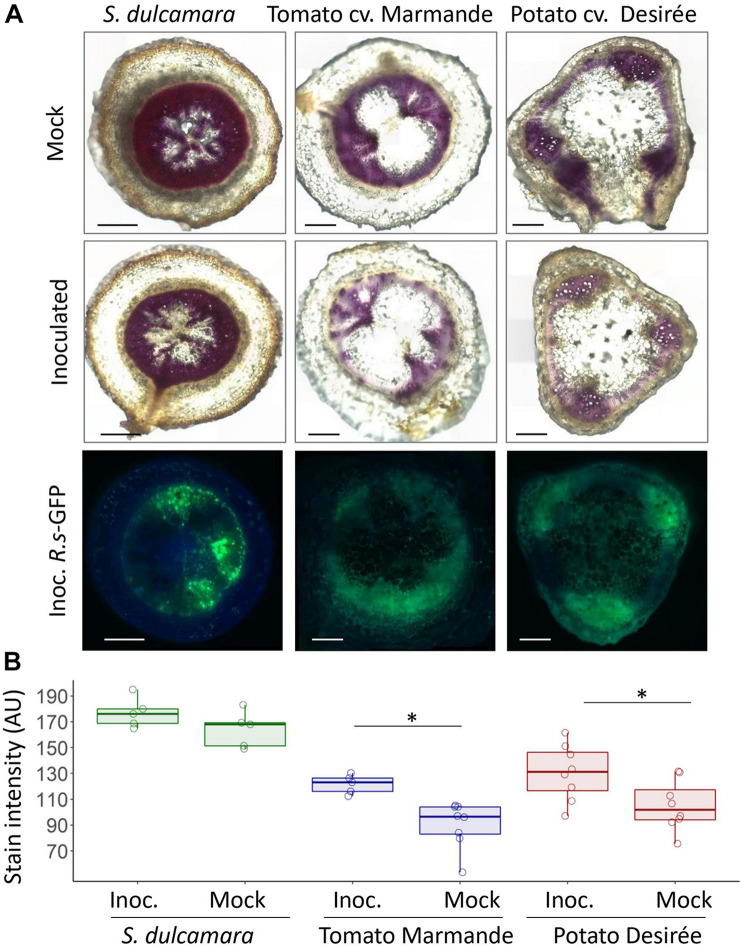
Lignification of *S. dulcamara*, Tomato cv. Marmande and *S. tuberosum* cv. Desirée tissues upon *R. solanacearum* infection. **(A)** Representative composed images of *S. dulcamara*, tomato, and potato taproot transversal sections obtained 9 days after inoculation with *R. solanacearum-*GFP or mock treatment. First and second row: microscope bright-field images after lignin staining with phloroglucinol HCl (magenta colouration). Third row: fluorescence microscopy images after inoculation to assess the extent of bacterial colonisation. Images were obtained using a Leica DM6 microscope. Scale bars indicate 0.5 mm. **(B)** Quantification of the phloroglucinol HCL stain -indicative of lignin content- in the vascular area from the images shown in A performed with the ImageJ software. ^∗^indicates statistical differences (*p*-value < 0.05; *T*-student significant test α = 0.05).

The colonisation pattern of *R. solanacearum* in *S. dulcamara* compared with tomato cv. Marmande suggested that the former may contain vascular structures or components that make bacterial movement difficult. Lignin is one of the main components of the secondary plant cell wall, and it has been described to play an important role as a structural defence mechanism in resistant tomato varieties against *R. solanacearum* (Nakaho et al., 2000; Ishihara et al., 2012; Kashyap et al., 2021). Therefore, we tested whether *S. dulcamara* xylem vessels presented differential lignin accumulation in their cell walls compared with susceptible tomato and potato plants. Taproot sections obtained 9 days after mock or soil inoculation with the *R. solanacearum* GFP reporter strain were stained with phloroglucinol HCl to identify lignified structures. This revealed constitutive and conspicuous lignification of the *S. dulcamara* vasculature, whereas, in susceptible tomato and potato plants, the parenchyma cells surrounding the vascular cylinder did not appear lignified (Figure 5A). In addition, while lignification remained stable in *S. dulcamara* after *R. solanacearum* infection, both tomato and Desirée plants showed a significant decrease in lignin accumulation upon *R. solanacearum* infection (Figure 5A), as previously described (Kashyap et al., 2021). To avoid the effect of lower bacterial concentrations usually found in *S. dulcamara* tissues, plants that contained comparable bacterial colonisation, as assessed by bacterial GFP fluorescence, were used for staining (Figure 5A lower panel). Quantification of the lignin stain intensity in mock and infected plants clearly confirmed a decrease in inoculated tomato and potato that was not observed in *S. dulcamara* plants (Figure 5B). The same results were observed after lignin staining from samples obtained at 6 dpi (Supplementary Figure 3), when bacterial colonisation was still low (Supplementary Figure 3A lower panel).

We have recently described (Kashyap et al., 2021) that suberin plays an important role in tomato resistance to bacterial wilt. To evaluate if this compound had an effect on the *S. dulcamara* restriction to *R. solanacearum* colonisation, we also stained inoculated or mock-treated stem sections with Sudan IV, which binds to the aliphatic domain of suberin to produce a reddish-brown colouration. No detectable increase in the accumulation of suberin was observed in *S. dulcamara* or in the susceptible plants after infection or mock treatment, as shown in Supplementary Figure 6.

In sum, *S. dulcamara* presented a constitutive accumulation of lignin in the xylem vessels and surrounding parenchyma that was not reduced upon pathogen infection as observed in susceptible tomato and potato, which may explain a higher restriction of bacterial colonisation in this species.

## Discussion

### *Solanum dulcamara* Shows Partial Resistance to Bacterial Wilt

It has been established that plants display two main types of defence against pathogens: resistance, which is the ability of the host to limit pathogen multiplication; and tolerance, defined as the ability of the host to reduce the negative effects of infection (Clarke, 1986; Pagán and García-Arenal, 2020). However, the term tolerance has often been used incorrectly to describe partial levels of plant resistance. To differentiate bona fide tolerance from partial resistance a key aspect is that tolerance implies that the plant shows less symptoms or yield effects at equivalent levels of pathogen loads (Pagán and García-Arenal, 2020). According to these definitions, *S. dulcamara* shows some degree of resistance to bacterial wilt and no tolerance to the pathogen. A clear proof that tolerance does not play a role in the response of *S. dulcamara* to *R. solanacearum* is that all direct and indirect quantifications *R. solanacearum* inside *S. dulacamara* plants are comparable with those observed in tomato plants showing similar symptoms (Figures 1E,F, 2 and Supplementary Figures 2, 3, 6). The only exception to this is totally wilted plants, where bacterial populations declined, likely due to the lack of humidity in dry dead tissues (e.g., plant F, Figure 2A).

Several observations support that *S. dulcamara* plants display partial resistance to bacterial wilt (Table 1). First, irrespective of the inoculation method used, *S. dulcamara* showed delayed symptomatology (Figures 1A,B), delayed stem colonisation (Figures 1C,D), and slightly delayed root colonisation (Supplementary Figure 4). These phenotypes are similar but less pronounced than those observed in resistant tomato cv. Hawaii 7996 (Table 1 and Planas-Marquès et al., 2020). Further proofs of this are that an important proportion of *S. dulcamara* plants remained asymptomatic when tomatoes were completely wilted and that stem inoculation and large numbers had to be used to obtain enough plants at advanced disease stages to compare with susceptible tomatoes.

**TABLE 1:** Schematic comparison of the interaction at different levels between *R. solanacearum* and a susceptible tomato, *S. dulcamara*, and a resistant tomato.

	Susceptible tomato	*S. dulcamara*	Resistant tomato
Disease symptoms	+++	++	±
Bacterial levels in roots	+++	++	+
Bacterial levels in stems	+++	++	+
Bacterial vertical spread	+++	++	+
Bacterial horizontal spread	+++	++	+
Symptoms at lower temp.	++	±	NT
Structural reinforcements	±	++	+++

*±: few or inexistent; +: low levels; ++: intermediate levels; +++: high levels; NT: not tested.*

Second, *S. dulcamara* restricted *R. solanacearum* vertical movement in the stem. A luminescent *R. solanacearum* reporter strain was able to entirely colonise susceptible tomato, while in *S. dulcamara* plants the upper stem displayed less pathogen colonisation (Figures 2, 3A). In accordance with this, it has been described in *S. dulcamara* that symptomatology and pathogen presence was restricted only to shoots directly inoculated, indicating slow or no spreading of the bacterium throughout the plant (Wenneker et al., 1999). We previously reported similar behaviour, although clearly more apparent (Table 1) in resistant tomatoes (Planas-Marquès et al., 2020).

Third, bacterial movement between xylem vessels was also limited in *S. dulcamara* compared with susceptible tomatoes (Figure 3B). This could explain the stem colonisation delay observed, as *S. dulcamara* restricts *R. solanacearum* to specific xylem vessels, while others remain pathogen-free, a behaviour also reported for the resistant tomato (Planas-Marquès et al., 2020).

Taken together, our results confirm previous studies that reported *S. dulcamara* as partially resistant to bacterial wilt, although it was misleadingly described as tolerance. Discrepancies amongst previous reports where infection rates varied from 100 to 66% and 13 to 19% (Wenneker et al., 1999; Álvarez et al., 2008b; Jacobs et al., 2013) can be explained by the different inoculation methods used, by different assay conditions (e.g., temperature and inoculum), and/or by genetic differences amongst the plant accessions used.

### *Solanum dulcamara* Carries Latent *R. solanacearum* Infections at 20°C

Three conditions are required for the establishment and development of plant diseases: a virulent pathogen, a susceptible host, and permissive environmental conditions (McNew, 1960). We thus explored the behaviour of *S. dulcamara* resistance to bacterial wilt when plants are grown and inoculated at lower temperatures. A decrease in temperature resulted in delayed symptom appearance and bacterial colonisation in both the susceptible hosts and in *S. dulcamara* (Figure 4) and the difference in resistance between them was maintained. This indicates that the ability of a pathogen to cause disease is compromised at a lower temperature, as has been described for many pathosystems. Thus, in these conditions, *S. dulcamara* plants displayed a stronger resistance to the disease, as all plants survived a month after inoculation and only very few of them showed minor wilting symptoms (Figure 4A and Table 1), but they all carried asymptomatic (latent) infections (Figure 4B).

Tolerance to disease, i.e., the ability to keep high bacterial levels without showing symptoms, has been proposed as a key trait for plants to act as reservoir hosts, providing a source of pathogen inoculum that spreads when environmental conditions become appropriate (Roberts and Heesterbeek, 2020). Based on our findings with *S. dulcamara* (Table 1), we propose that resistance, i.e., limiting pathogen colonisation, could also enable plants to act as reservoirs. Intermediate resistance would be required in this case for two reasons: first, it would allow a limited amount of pathogen to colonise and survive under unfavourable environmental conditions, such as winter temperatures, as latent infections inside the plant, and second, when environmental conditions favour disease (high temperature in our case), the pathogen could overcome resistance in some plants, multiplying to high numbers and spreading to other hosts. These two conditions could not take place if plants were either fully resistant or tolerant. This theory is supported by the original description of *S. dulcamara* as a symptomless *R. solanacearum* carrier in the wild (Olsson, 1976; Hayward, 1991), and studies show that environmental conditions can break resistance to the disease. For instance, in eucalypt, *R. solanacearum* usually behaves as a latent colonist, and only in the presence of other stressing factors the pathogen is able to proliferate and cause disease (Coutinho and Wingfield, 2017).

### Constitutive Xylem Lignification in an *S. dulcamara* Is Likely Responsible for Its Resistance to *R. solanacearum*

Observation of *S. dulcamara* stem transversal sections indicated a highly lignified xylem compared with susceptible tomato and potato varieties (Figure 5 and Supplementary Figure 5). This is in accordance with previous reports that lignin biosynthesis genes were upregulated in the bacterial wilt resistant tomato variety LS-89 upon *R. solanacearum* infection (Ishihara et al., 2012). Furthermore, we have recently shown striking differences in lignin composition between susceptible (Marmande) and resistant (Hawaii 7996) tomatoes, which indicate that the properties of paravascular lignin may be key for resistance to bacterial wilt (Kashyap et al., 2021).

Interestingly, *S. dulcamara* lignification was already high in mock-treated plants and was not affected by infection (Figure 5 and Supplementary Figure 5), whereas susceptible tomato and potato plants reduced their lignin content significantly both at 6 and 9 dpi upon *R. solanacearum* inoculation. This constitutive lignification and the irrelevance of suberin components, whose levels are comparable with susceptible plants (Supplementary Figure 6), are key differences in the factors controlling *S. dulcamara* resistance compared with tomato H7996, where suberin components play a major role (Kashyap et al., 2021). The fact that *S. dulcamara* is a perennial, with the ensuing secondary growth present in these plants (Caldwell, 2016), may explain the high lignification of its tissues, which must be even more pronounced in wild plants- and that this phenomenon is not inducible like in the annual tomato plants.

The correlation observed between resistance to infection and the presence of cell wall reinforcements both in resistant tomato and in the wild *S. dulcamara* plants indicates that lignification hinders *R. solanacearum* movement throughout the plant tissues and entry in the xylem vessels. This would explain the delay in symptom appearance compared with susceptible tomatoes (Figures 1A,B) and also account for the low bacterial content in inoculated plants that remained healthy (Figures 1C–F). Microscopically, cell wall reinforcements could have a major contribution to the stronger bacterial restriction in specific xylem vessels and decreased spread to neighbouring parenchyma cells, as observed in *S. dulcamara* compared with tomato cv. Marmande (Figure 3). Restriction of *R. solanacearum* infection to primary xylem vessels while secondary xylem vessels remain functional (Esau, 1977) could explain why *S. dulcamara* better survives the infection. Restricting pathogen movement is an important mechanism for resistance against *R. solanacearum* in tomato (Caldwell et al., 2017; Planas-Marquès et al., 2020) and potato (Cruz et al., 2014) that is also conserved in grapevine against *Xylella fastidiosa* (Chatterjee et al., 2008).

In summary, strong preexisting lignified xylem vessels present in *S. dulcamara* are likely the factor that supports its resistance to *R. solanacearum* and allows it to behave like a reservoir host. The generation of *S. dulcamara* mutants in lignin biosynthesis genes would be extremely useful to confirm this hypothesis.

## Data Availability Statement

The original contributions presented in the study are included in the article/Supplementary Material, further inquiries can be directed to the corresponding author.

## Author Contributions

MV, PS, NC, and AK conceived and designed the work. PS, AK, RP-J, and BD performed the experiments and statistical analyses. MV and NC provided reagents and materials. PS, RP-J, and MV analysed the results and edited the figures. MV, PS, and NC wrote the manuscript. All authors have made a substantial, direct and intellectual contribution to the work, and approved it for publication.

## Conflict of Interest

The authors declare that the research was conducted in the absence of any commercial or financial relationships that could be construed as a potential conflict of interest.

## Publisher’s Note

All claims expressed in this article are solely those of the authors and do not necessarily represent those of their affiliated organizations, or those of the publisher, the editors and the reviewers. Any product that may be evaluated in this article, or claim that may be made by its manufacturer, is not guaranteed or endorsed by the publisher.
