# Supplementary material for: The Bacterial Wilt Reservoir Host Solanum dulcamara Shows Resistance to Ralstonia solanacearum Infection
Source: Front Plant Sci. 2021 Nov 10;12:755708. doi: 10.3389/fpls.2021.755708 (PMC8636001; doi:10.3389/fpls.2021.755708)
Supplement: Supplementary file 1 [file Data_Sheet_1.PDF]

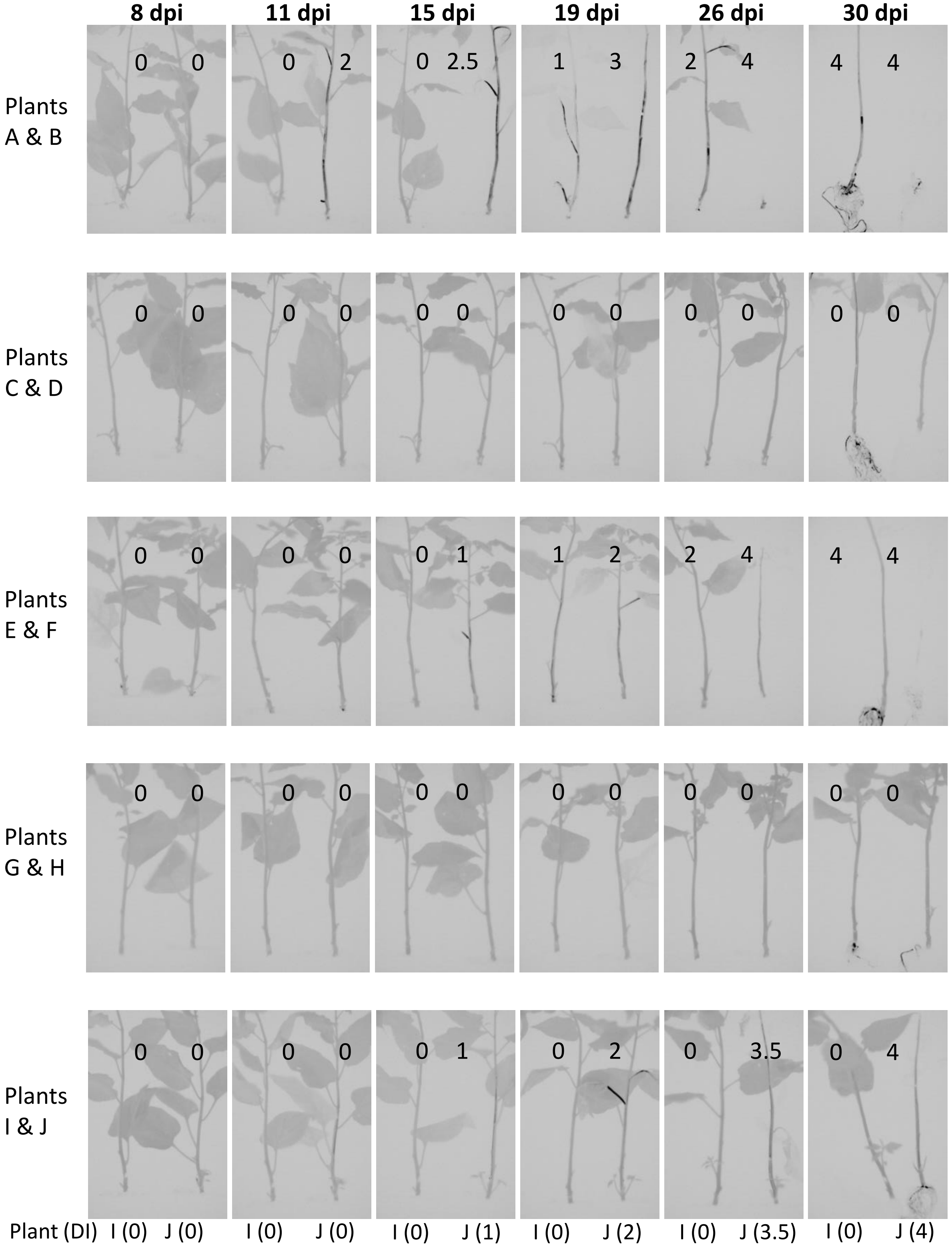

FIGURE S1. Bacterial colonization in *S. dulcamara* at different time points. Representative pictures of whole *S. dulcamara* plants taken at 8 to 30 days after root inoculation with a *R. solanacearum* luminescent strain. Numbers in the pictures indicate the disease index when they were taken and letters identify each individual.

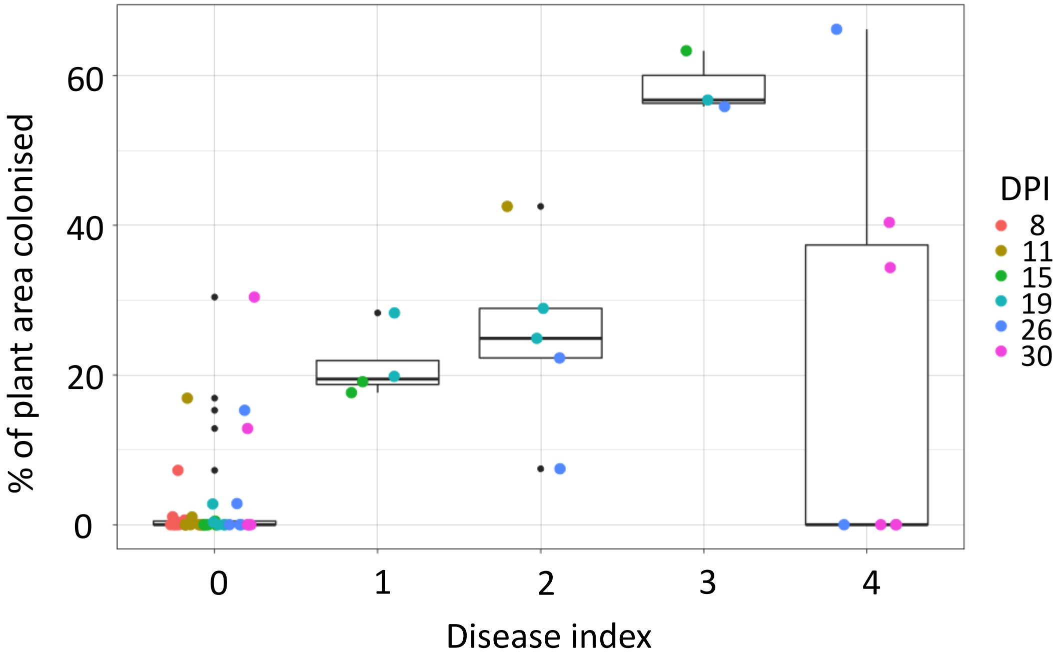

FIGURE S2. Nondisruptive quantification over time of the *S. dulcamara* stem area colonised by *R. solanacearum* after inoculation. The total plant surface was measured and the infected area was quantified by measuring the dark area (bacterial luminescence) from pictures shown in figure S1, which had been root inoculated with a luminescent *R. solanacearum* strain. Images were processed using Fiji software (U. S. National Institutes of Health). Each dot corresponds to the colonised area of a single plant at a precise day after inoculation (DPI). Boxplot central rectangles span the first quartile to the third quartile (interquartile range), horizontal lines inside rectangles show the median, black spots indicate suspected outliers, whiskers above and below the box show either the locations of the minimum and maximum in the absence of suspected outlying data.

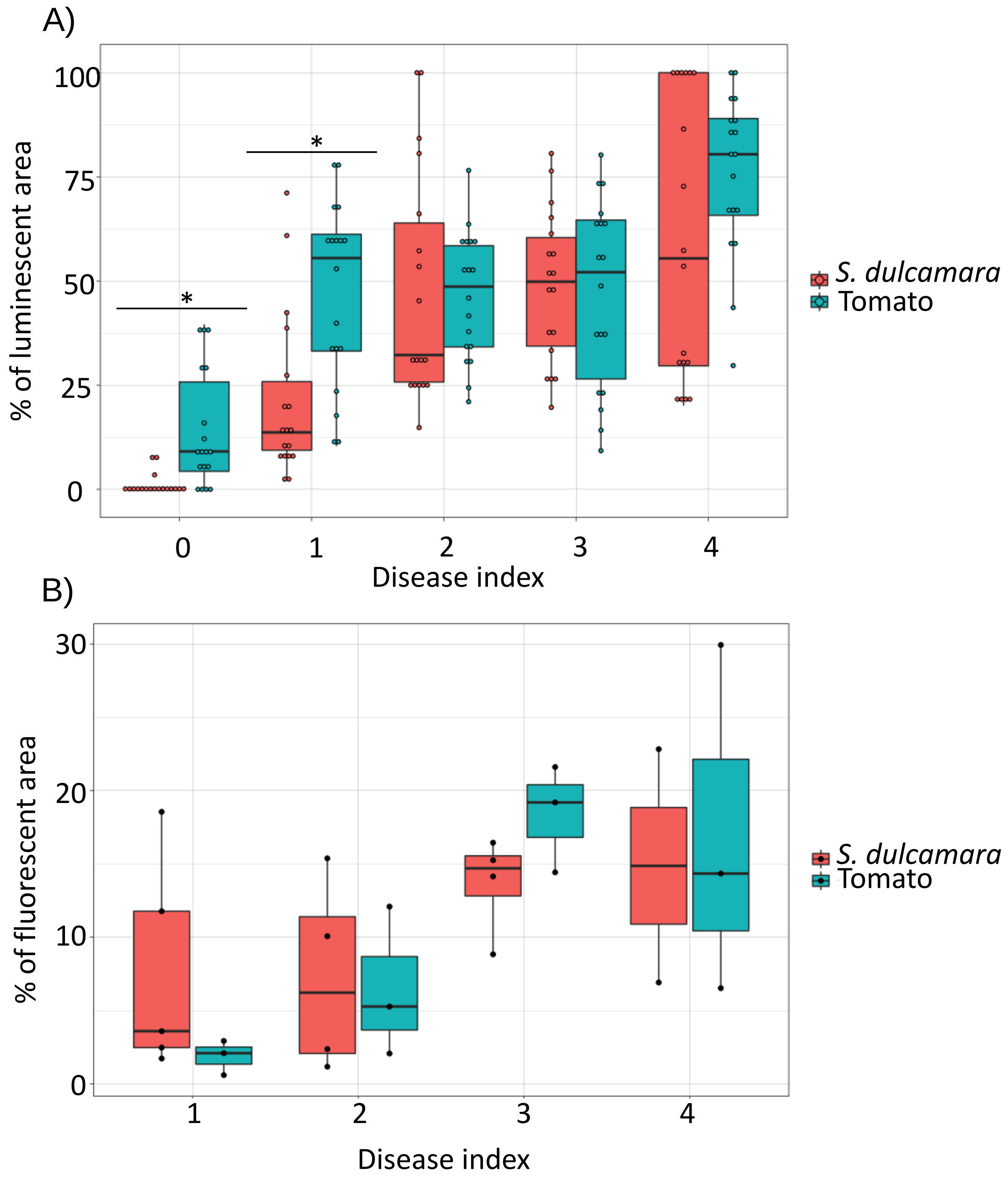

FIGURE S3. Quantification of *R. solanacearum*-infectied area in *S. dulcamara* and tomato stems. Images showing luminescence or fluorescence in stem transversal sections of *S. dulcamara* and tomato cv. Marmande plants (like those shown in figure 3A and 3B) were quantified using the Fiji software (U. S. National Institutes of Health). The luminescent (A) and fluorescent (B) area is shown as percentage of the longitudinal or transversal stem area, respectively. Boxplot central rectangles span the first quartile to the third quartile (interquartile range), horizontal lines inside rectangles show the median, black spots indicate suspected outliers, whiskers above and below the box show either the locations of the minimum and maximum in the absence of suspected outlying data. * indicates statistical differences (*p value*<0.05, T-student significant test).

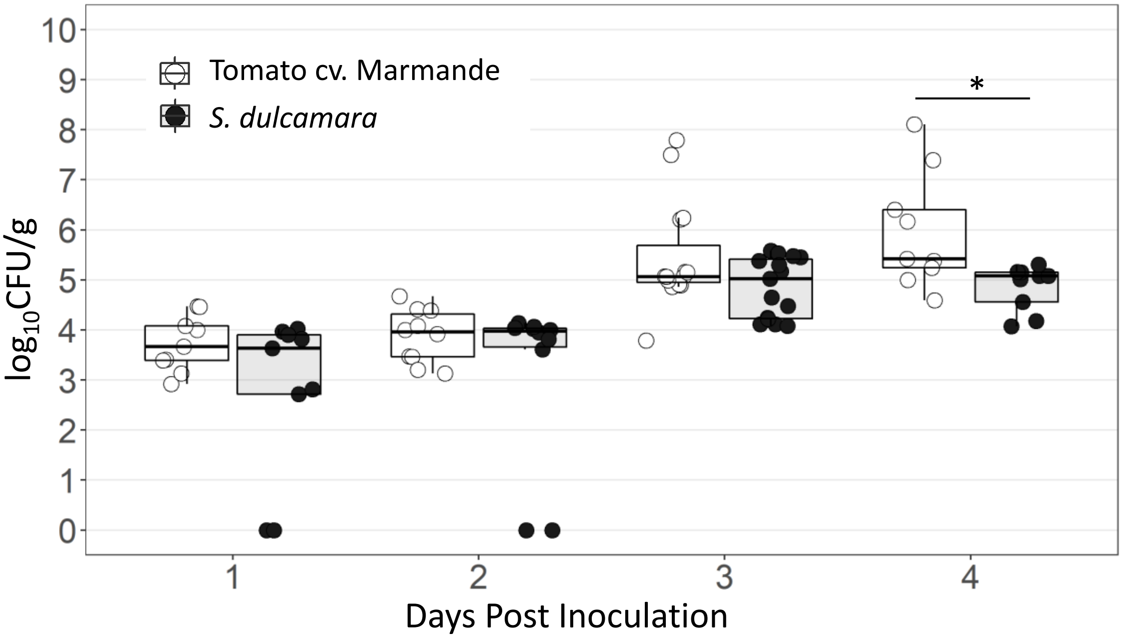

FIGURE S4. Early root colonisation of *S. dulcamara* and susceptible tomato by *R. solanacearum*. Bacterial concentrations in *S. dulcamara* (black dots) and tomato cv. Marmande (white dots) sampled from the root 1-2 cm below the collar 1 to 4 days after root inoculation with a *R. solanacearum* luminescent strain. Bacterial counts were calculated from tissue luminescence and are expressed as log CFUs·g^{-1} tissue. n=8 to 10 plants per assayed day. * indicates statistical differences (*p value*<0.05, T-student significant test).

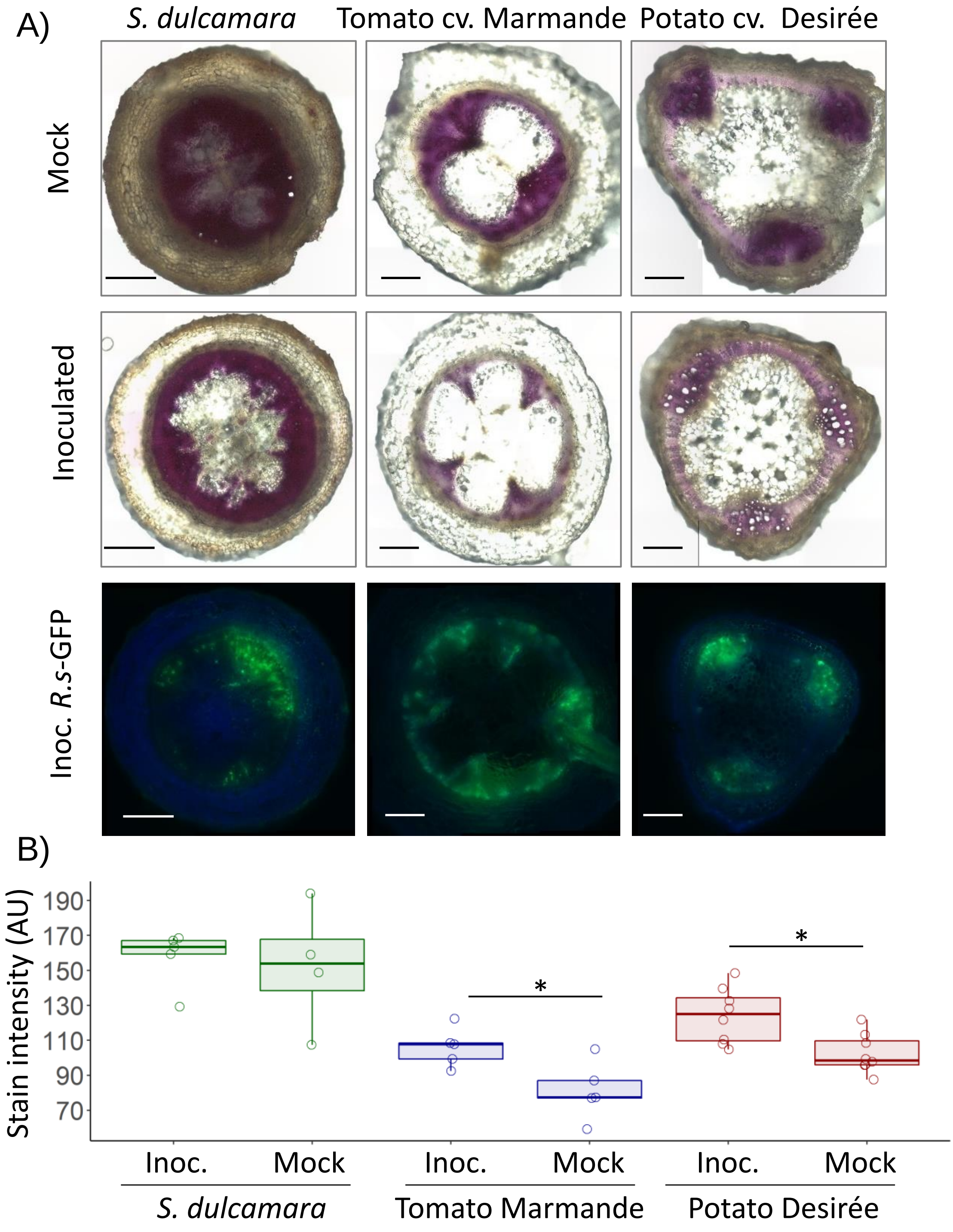

FIGURE S5. Plant lignification after *R. solanacearum* infection. A) Representative composed images from Leica DM6 microscope pictures of taproot transversal sections obtained 6 days after *R. solanacearum* inoculation. First and second row: samples stained with phloroglucinol HCl and observed under bright field. Third row: fluorescence microscope images of the same samples. Scale bars= 0.5 mm. B) Quantification of the phloroglucinol HCL stain from the images shown in A. * p-value < 0.05; T-student significant test α=0.05.

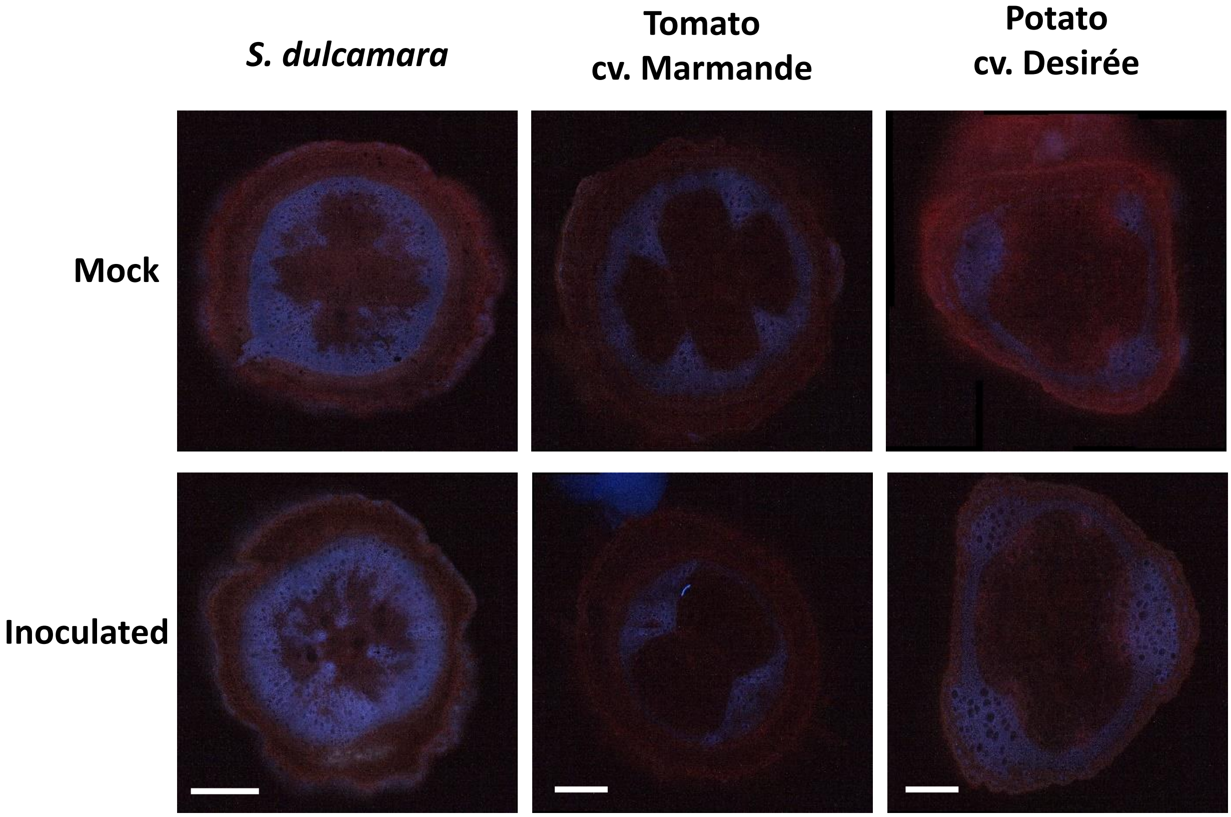

Figure S6. Suberin staining of *S. dulcamara*, *S.* lycopersicum cv. Marmande and *S. tuberosum* cv. Desirée. Observation of taproot transversal sections after sudan IV staining of mock-treated or *R. solanacearum*-inoculated plants. Images were obtained with a Leica DM6 upright microscope. The scale bar indicates 0.5 mm.
